# A Multimodal Hybrid Piezoelectric–Electromagnetic Vibration Energy Harvester Exploiting the First and Second Resonance Modes for Broadband Low-Frequency Applications

**DOI:** 10.3390/s26072092

**Published:** 2026-03-27

**Authors:** Dejan Shishkovski, Zlatko Petreski, Simona Domazetovska Markovska, Maja Anachkova, Damjan Pecioski, Anastasija Angjusheva Ignjatovska

**Affiliations:** Faculty of Mechanical Engineering-Skopje, Ss. Cyril and Methodius University in Skopje, 1000 Skopje, North Macedonia; zlatko.petreski@mf.edu.mk (Z.P.); simona.domazetovska@mf.edu.mk (S.D.M.); maja.anachkova@mf.edu.mk (M.A.); damjan.pecioski@mf.edu.mk (D.P.); anastasija.ignjatovska@mf.edu.mk (A.A.I.)

**Keywords:** energy harvesting, hybrid piezoelectric–electromagnetic harvester, multimodal resonance, broadband operation

## Abstract

The increasing demand for autonomous wireless sensors in Internet of Things (IoT) applications has intensified research on vibration energy harvesting, particularly in the low-frequency range where ambient vibrations are most prevalent. However, most vibration energy harvesters operate efficiently only at a single resonance mode, resulting in a narrow operational bandwidth and pronounced performance degradation under frequency detuning. To address this limitation, this paper proposes a multimodal hybrid piezoelectric–electromagnetic vibration energy harvester that exploits both the first and second resonance modes of a cantilever-based structure to achieve broadband low-frequency operation. The design is guided by the complementary utilization of strain-dominated and velocity-dominated regions associated with different vibration modes. Numerical modeling and finite element simulations are employed to investigate the influence of mass distribution, deformation characteristics, and relative velocity on energy conversion performance. A secondary cantilever carrying the electromagnetic coil is introduced to enhance the relative motion between the coil and the magnetic field, thereby extending the effective operational bandwidth. The experimental results demonstrate increased harvested power, improved energy conversion efficiency, and a significantly broadened effective frequency range compared to conventional single-mode piezoelectric and electromagnetic energy harvesters.

## 1. Introduction

The rapid expansion of the Internet of Things (IoT) and wireless sensor networks (WSNs) has led to a growing demand for autonomous sensor nodes capable of long-term operation without periodic battery replacement or maintenance. In many industrial, infrastructural, and mechanical systems, sensors are deployed in remote or hard-to-access locations, where conventional power solutions become impractical, costly, or infeasible [1,2]. These constraints have motivated extensive research into energy harvesting (EH) technologies that can convert ambient mechanical vibrations into usable electrical energy, enabling self-powered and maintenance-free sensor systems [3,4].

Early foundational studies demonstrated that low-level ambient vibrations, typically present in industrial machinery, can be effectively exploited as a power source for wireless nodes, particularly at frequencies below 100 Hz [5]. Low-frequency vibrations in the context of electromechanical energy harvesting are commonly categorized into distinct regimes depending on the application domain. Civil and structural systems often exhibit sub-Hz to a few-Hz dynamics, whereas rotating industrial machinery typically operates within the range of 5–60 Hz, depending on rotational speed and imbalance conditions.

In this work, the term “low-frequency” refers specifically to the vibration range relevant to industrial rotating machinery and distributed mechanical systems, typically below 100 Hz and more specifically within 5–60 Hz. The proposed system is not intended for ultra-low-frequency (<1 Hz) civil structural applications, which typically require different design strategies such as inertial amplification, frequency up-conversion, or large-scale mechanical tuning.

Recent studies have demonstrated effective energy harvesting under sub-Hz structural excitation using specialized amplification mechanisms and large-displacement configurations [6]. Such ultra-low-frequency harvesters are primarily optimized for civil and large-scale structural monitoring applications characterized by very low excitation frequencies and large displacement amplitudes.

In contrast, the present work targets the persistent mechanical vibration spectra encountered in rotating machinery, industrial equipment, and electromechanical assemblies, where compact multimodal coupling and controlled detuning represent more suitable design strategies.

Among the available mechanisms, piezoelectric (PZT) and electromagnetic (EM) transduction have emerged as the most widely investigated [7]. PZT harvesters are particularly attractive due to their high energy density and suitability for micro-scale implementations [8,9,10]. However, their performance is typically maximized only in narrow frequency bands and is strongly dependent on strain localization [11]. Electromagnetic harvesters, on the other hand, exhibit favorable performance in velocity-dominated vibration regimes and provide robust power generation under larger amplitudes, but often suffer from lower power density at low frequencies.

To overcome the limitations of single-transduction mechanisms, hybrid energy harvesting systems combining piezoelectric (PZT) and electromagnetic (EM) principles have been proposed [12,13]. Such configurations exploit complementary physical domains—strain-based and velocity-based energy conversion—to enhance output power and improve robustness against frequency variations [14,15]. Nevertheless, many reported hybrid harvesters remain limited to a single dominant resonance, resulting in narrow operational bandwidths [16,17]. Since real-world excitation frequencies are often time-varying and unpredictable, achieving broadband operation remains a critical challenge [18,19].

Various strategies have been explored to address this, including dual-resonance structures and nonlinear mechanisms [3,4]. Dual-resonance EM harvesters exploit coupled resonant structures to extend the operational range, while spring-less EM harvesters employing nonlinear magnetic stiffness have been shown to significantly extend bandwidth while maintaining high mechanical quality factors [5]. Furthermore, analytical studies emphasize that maximum power extraction occurs when electrical and mechanical damping are optimally matched [20]. Beyond mechanical design, recent research has successfully applied artificial intelligence (AI) and optimization-based approaches to identify optimal harvester parameters under varying scenarios [11,21]. To position the proposed system within the current state of the art, Table 1 summarizes representative multimodal and dual-resonance vibration energy harvesters reported in the literature. Most broadband designs rely on independent resonant modes, nonlinear stiffness mechanisms, or multi-degree-of-freedom architectures to extend the half-power bandwidth.

As shown in Table 2, previously reported broadband harvesters typically achieve bandwidth extension factors on the order of two times relative to single-mode resonant structures. In contrast, the present work introduces controlled detuning between mechanically coupled cantilevers, generating modal interaction and a beating phenomenon. When evaluated using a practical IoT operational threshold (P ≥ 0.5 mW), the proposed system achieves approximately a two- to three-fold extension of the effective usable frequency range in the low-frequency regime below 100 Hz. Despite these advances, two key challenges remain. First, the spatial distribution of strain and velocity along a vibrating structure differs significantly between vibration modes [24,25,26,27], yet many designs fail to account for this mode-dependent energy localization. Second, the integration of multiple transduction mechanisms is often performed without a clear physical rationale for transducer placement.

In this work, a multimodal hybrid piezoelectric–electromagnetic vibration energy harvester is proposed, explicitly exploiting the first and second resonance modes of a cantilever-based structure. The design is based on the concept of mode-specific energy zones, where strain-dominated regions are targeted for piezoelectric transduction and velocity-dominated regions for electromagnetic conversion. A dual-mass tuning strategy is employed to independently tailor the dynamic response of the first and second modes, enabling enhanced energy localization and improved broadband performance in the low-frequency range. The proposed concept is investigated through numerical modeling and finite element analysis (FEA) to identify the spatial distribution of strain and velocity for different vibration modes and mass configurations. Experimental validation is carried out using an electrodynamic shaker and a real rotating-machine test bench, demonstrating enhanced power output and extended operational bandwidth compared to single-transduction configurations. Finally, the developed harvester is integrated into an autonomous IoT sensor node for vibration monitoring, highlighting the practical applicability of the proposed approach.

In industrial environments, although centralized electrical power is often available, distributed sensing nodes are frequently installed at locations where wired power delivery is impractical, costly, or undesirable. Examples include rotating or vibrating components, confined mechanical housings, safety-isolated structures, and large distributed machinery where extensive cable routing increases installation complexity and maintenance costs. In such scenarios, vibration energy harvesting does not aim to replace grid power, but rather to enable autonomous or maintenance-reduced wireless sensing. By reducing battery replacement intervals or eliminating wiring requirements, energy harvesters can significantly decrease operational downtime and total cost of ownership in large-scale condition-monitoring deployments. Therefore, the proposed hybrid energy harvester targets application-specific environments characterized by persistent low-frequency vibrations and limited accessibility, such as industrial rotating machinery, wind turbine towers, structural monitoring of bridges, and remote mechanical assets.

The novelty of this work lies in the physically guided exploitation of mode-dependent energy localization within a multimodal hybrid vibration energy harvesting system. The main contributions of this work can be summarized as follows:

1. A multimodal hybrid energy harvesting architecture that simultaneously exploits the first and second resonance modes for broadband low-frequency operation.
2. A dual-mass tuning methodology that enables the controlled redistribution of kinetic and potential energy along the cantilever structure.
3. An experimental demonstration of broadband vibration energy harvesting with improved performance compared to standalone piezoelectric and electromagnetic harvesters.
4. Integration and validation of the proposed harvester in an autonomous sensor system for real-world vibration monitoring applications.

## 2. System Architecture and Design Concept

**Figure 1:**
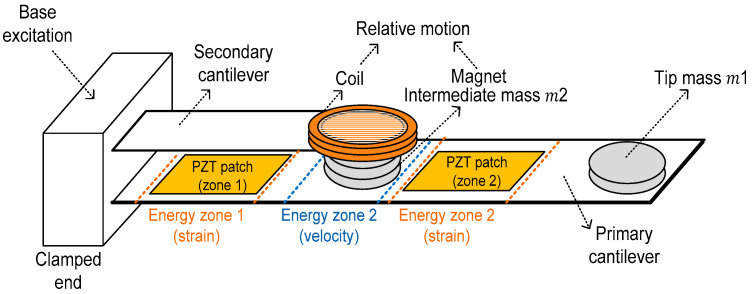
Conceptual schematic of the proposed multimodal hybrid piezoelectric–electromagnetic vibration energy harvesting system. Vibrations are introduced as base excitation at the clamped end of the primary cantilever. Dual-mass tuning (*m*_1_, *m*_2_) enables controlled exploitation of the first and second resonance modes. Piezoelectric patches harvest strain-dominated energy in Energy Zones 1 and 2, while the electromagnetic transducer exploits velocity-dominated energy through relative motion between the magnet and the coil mounted on a secondary cantilever.

The proposed energy harvesting system is based on a cantilever-type mechanical structure specifically designed to exploit multiple vibration modes for broadband low-frequency energy harvesting. The architecture integrates piezoelectric and electromagnetic transducers within a single mechanical framework, enabling simultaneous conversion of strain-dominated and velocity-dominated vibrational energy. Figure 1 presents the conceptual schematic of the proposed system, highlighting the dual-mass tuning strategy, energy zones, and hybrid piezoelectric–electromagnetic transduction.

### 2.1. Primary Cantilever and Multimodal Tuning Strategy

The primary mechanical structure consists of an elastic cantilever beam clamped at one end and free at the other. To tailor the dynamic behavior of the system and align its resonance frequencies with typical industrial vibration spectra, a dual-mass tuning approach is employed. Two concentrated masses, denoted as *m*_1_ and *m*_2_, are strategically placed along the cantilever to independently influence the first and second vibration modes. The first mass *m*_1_, positioned at the free end of the cantilever, primarily affects the first natural frequency by increasing the effective inertia and lowering the fundamental resonance into the low-frequency range below 10 Hz. This tuning is particularly relevant for harvesting energy from slow structural oscillations commonly encountered in industrial machinery and infrastructure. The second mass *m*_2_ is placed in the region of maximum displacement and velocity associated with the second vibration mode. Unlike *m*_1_, this mass has only a minor influence on the first mode but significantly reduces the second natural frequency and modifies the corresponding mode shape. This configuration enables the controlled redistribution of kinetic energy and creates a region characterized by high effective velocities and nearly linear motion, which is particularly favorable for electromagnetic energy conversion.

### 2.2. Definition of Energy Zones and Transducer Placement

Based on detailed modal and energy analysis, the cantilever structure is divided into distinct energy zones corresponding to dominant deformation and velocity characteristics. An energy zone near the clamped boundary exhibits maximum bending stresses and strain energy, making it optimal for the integration of piezoelectric transducers. A piezoelectric patch is therefore bonded in this region to harvest energy from the strain-dominated first vibration mode.

A second energy zone is defined around the location of maximum displacement and effective velocity during the second vibration mode. This region is characterized by predominantly translational motion with minimal lateral deflection, enabling stable electromagnetic energy harvesting without mechanical interference. A permanent magnet is mounted at this location and serves as the moving element of the electromagnetic transducer.

This spatial separation of energy zones allows each transduction mechanism to operate under conditions best suited to its physical principle, thereby maximizing the overall energy conversion efficiency of the system.

### 2.3. Secondary Cantilever and Controlled Resonance Detuning

To further enhance electromagnetic energy conversion, a secondary cantilever is introduced and rigidly attached to the base of the primary structure. The electromagnetic coil is mounted on the secondary cantilever, while the permanent magnet remains attached to the primary cantilever. The secondary cantilever is dimensioned such that its first natural frequency lies close to, but not exactly coincident with, the second natural frequency of the primary cantilever.

This intentional detuning results in a small frequency mismatch between the two oscillating structures, leading to a beating phenomenon when excited near resonance. The resulting periodic amplification of relative velocity between the magnet and the coil significantly enhances electromagnetic energy harvesting and contributes to a widening of the effective operational frequency range.

### 2.4. Hybrid Multiphysics Energy Conversion Concept

The combined system operates as a hybrid multiphysics energy harvester in which mechanical vibrations are converted into electrical energy through two complementary pathways. The piezoelectric transducer efficiently harvests energy from high-strain regions during the first vibration mode, while the electromagnetic transducer dominates energy conversion during the second vibration mode due to increased relative velocities.

By deliberately exploiting the first and second resonance modes and integrating a secondary oscillatory structure, the proposed architecture achieves broadband low-frequency energy harvesting under realistic excitation conditions. This multimodal hybrid design forms the foundation for the experimental investigations and performance evaluation presented in the following sections.

## 3. Multimodal Dynamic Behavior and Resonance Tuning

### 3.1. Modal Characteristics of the Untuned Cantilever

The dynamic behavior of the proposed energy harvesting system is governed by the modal properties of the cantilever structure. For the baseline configuration without added masses, the primary cantilever exhibits a first natural frequency in the range of approximately 18–20 Hz and a second natural frequency above 110 Hz. While such modal characteristics are typical for slender cantilever beams, they are poorly aligned with the dominant vibration spectra encountered in industrial and infrastructural environments, where excitation frequencies are mostly found below 100 Hz.

Moreover, in the untuned configuration, the usable energy is concentrated within a narrow frequency band around the fundamental resonance, which significantly limits the effectiveness of single-mode energy harvesting under variable excitation conditions. These limitations motivate the need for deliberate modal tuning and multimodal exploitation in order to extend the operational bandwidth of the system.

To clarify the tunability of the resonance frequencies, the natural frequency of each cantilever can be approximated using a simplified single-degree-of-freedom representation:

(1) $$f_{n}=\frac{1}{2\pi }\sqrt{\frac{K_{eff}}{m_{eff}}}$$

where $K_{eff}$ denotes the effective stiffness and $m_{eff}$ the effective modal mass.

For a uniform Euler–Bernoulli cantilever beam, the fundamental frequency scales approximately as:

(2) $$f_{n}\propto \;\frac{1}{L^{2}}\sqrt{\frac{EI}{\rho A}}$$

This indicates that resonance frequencies can be tuned through tip mass variation, mass ratio adjustment, or geometric scaling of beam dimensions. Increasing tip mass reduces the resonance frequency, while increasing stiffness (e.g., thickness) shifts it upward. Controlled detuning between the primary and secondary cantilevers can therefore be achieved by selecting appropriate mass ratios *m*_1_/*m*_2_, enabling adaptation to application-specific vibration spectra.

The finite element model was constructed using the same geometric dimensions, material properties, and tip mass configurations as the experimental prototype. Minor deviations between simulated and experimental resonance frequencies are attributed to manufacturing tolerances, assembly imperfections, and additional damping effects not fully captured in the numerical model.

In numerical parametric analysis, discrete mass values of 0, 0.025, 0.05, 0.075, and 0.1 kg were considered to systematically evaluate the influence of tip mass variation on the modal behavior. The 0.1 kg value represents one of the discretized sweep parameters used for trend analysis. In the experimental prototype, the attached masses were implemented using commercially available permanent magnets with individual masses of 47 g, resulting in a combined mass of approximately 94 g. The ≈6% difference between the simulated 0.1 kg configuration and the experimentally realized 94 g configuration does not significantly affect the resonance frequencies or the dual-resonance interaction mechanism. The selected experimental configuration therefore represents a practical implementation consistent with the numerical trend analysis.

Material properties were modeled using nominal values for Young’s modulus and density, while damping was represented through an equivalent viscous damping approximation. Despite these simplifications, the good agreement between numerical predictions and experimental measurements confirms the validity of the modeling approach.

### 3.2. Effect of Tip Mass m_1_ on the First Resonance Mode

To shift the fundamental resonance toward lower frequencies, a concentrated mass *m*_1_ is introduced at the free end of the cantilever. This mass primarily increases the effective inertia of the system and has a pronounced influence on the first vibration mode, while its effect on higher modes remains comparatively limited.

As *m*_1_ is increased, the first natural frequency decreases significantly, enabling tuning into the low-frequency range below 10 Hz. For the selected mass value *m*_1_ = 94 g, the first natural frequency is reduced to approximately 6.5–7.5 Hz, corresponding to a decrease of more than 60% relative to the untuned configuration. In contrast, the second natural frequency is reduced by less than 25%, preserving sufficient separation between the first and second modes.

From an energy perspective, the introduction of *m*_1_ enhances bending deformation near the clamped boundary, leading to increased strain energy in this region. This behavior is particularly favorable for piezoelectric energy harvesting, as the piezoelectric transducer operates most efficiently under conditions of high mechanical strain. Consequently, the first vibration mode becomes the dominant contributor to piezoelectric energy conversion in the tuned low-frequency regime.

### 3.3. Effect of Intermediate Mass m_2_ on the Second Resonance Mode

While the tip mass *m*_1_ effectively tunes the first mode, it has only a limited impact on the second vibration mode. To address this, a second concentrated mass *m*_2_ is introduced at a location corresponding to the maximum displacement and effective velocity of the second mode.

The presence of *m*_2_ leads to a substantial reduction in the second natural frequency, while the first natural frequency remains nearly unchanged. For the configuration *m*_1_ = *m*_2_ = 94 g, the second resonance is shifted from values above 100 Hz to approximately 49–52 Hz, representing a reduction of nearly 50%. This frequency range closely matches typical vibration signatures of rotating machinery and electromechanical systems, making it highly relevant for practical applications.

In addition to frequency tuning, the mass *m*_2_ significantly modifies the second-mode shape. The nodal point is displaced along the cantilever, and a region of nearly linear translational motion is formed around the mass location. This region is characterized by high effective velocities and minimal lateral deflections, which is particularly advantageous for electromagnetic energy harvesting based on relative motion between a magnet and a coil.

### 3.4. Energy-Zone Separation and Mode-Specific Harvesting

**Figure 2:**
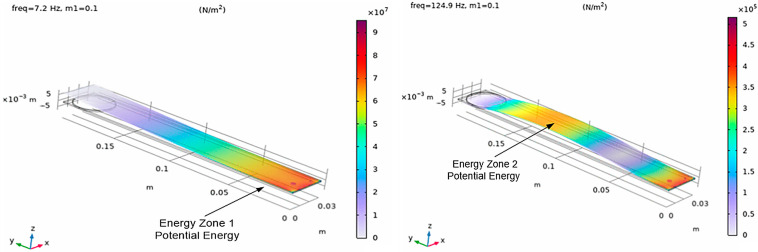
Distribution of normal stresses along the cantilever for the first and second vibration modes in the presence of an added tip mass *m*_1_, illustrating the mode-dependent localization of strain energy used for defining energy harvesting zones.

The combined effect of the dual-mass tuning strategy results in a clear spatial and modal separation of energy contributions along the cantilever. In the first vibration mode, the dominant energy component is strain energy concentrated near the clamped boundary, while the effective velocities remain relatively low along the remainder of the beam. As shown in Figure 2, the distribution of normal stresses along the cantilever strongly depends on the excited vibration mode, resulting in distinct strain-dominated regions that motivate the definition of separate energy zones for transducer integration.

In contrast, the second vibration mode exhibits moderate strain levels but significantly higher effective velocities in the region surrounding the mass *m*_2_.

**Figure 3:**
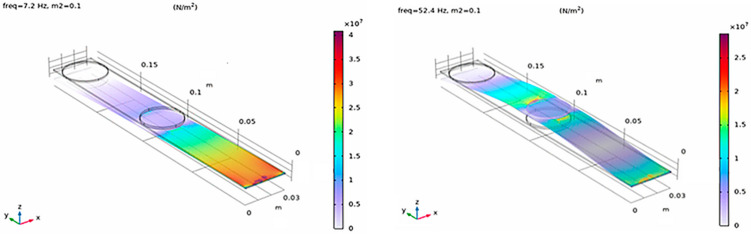
Distribution of normal stresses along the cantilever for the first and second vibration modes after introducing the second added mass *m*_2_, illustrating the redistribution of strain energy and the formation of a distinct energy zone associated with the second mode. Zones of maximum normal stresses for the first mode f_1_ = 7.2 Hz, and f_2_ = 52.4 Hz for the second mode with a second added mass *m*_2_ = 0.1 kg.

**Figure 4:**
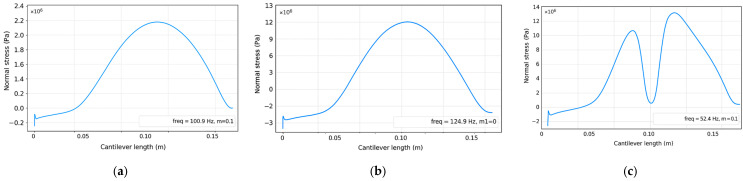
Comparison of the normal stress distribution along the cantilever for the second vibration mode: (**a**) without added mass, (**b**) with an added tip mass *m*_1_, and (**c**) with an added intermediate mass *m*_2_. The results illustrate the progressive localization and redistribution of strain-dominated regions that motivate multimodal and hybrid energy harvesting.

This behavior enables a deliberate allocation of transduction mechanisms to mode-specific energy zones. The piezoelectric transducer is positioned near the clamped end to exploit the strain-dominated first mode, whereas the electromagnetic transducer is integrated in the velocity-dominated region associated with the second mode. As a result, each transducer operates predominantly within the vibration mode that best matches its physical energy conversion principle. The introduction of the second added mass *m*_2_ leads to a pronounced redistribution of normal stresses along the cantilever, as shown in Figure 3, resulting in a well-defined strain-dominated region associated with the second vibration mode. The influence of the added masses on the redistribution of strain energy is further illustrated in Figure 4, which compares the stress distribution for the second vibration mode in three configurations: without added mass, with the tip mass *m*_1_, and with the intermediate mass *m*_2_.

**Figure 5:**
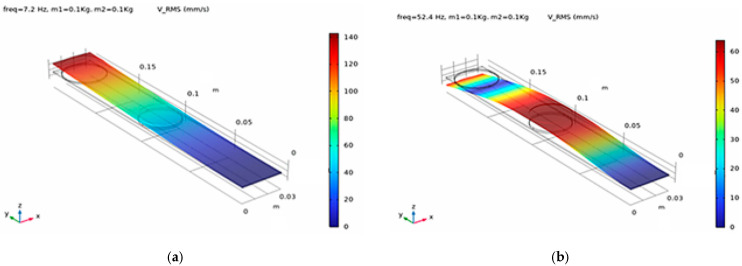
Distribution of vibration velocities along the cantilever for (**a**) the first vibration mode and (**b**) the second vibration mode after introducing the intermediate mass *m*_2_. The results highlight the formation of a velocity-dominated energy zone in the second mode, which is exploited for electromagnetic energy harvesting.

As shown in Figure 5, the second vibration mode exhibits significantly higher relative velocities compared to the first mode, resulting in a well-defined velocity-dominated energy zone suitable for electromagnetic energy conversion.

### 3.5. Multimodal Frequency Response and Bandwidth Implications

The tuned system exhibits two well-separated resonance peaks in the low-frequency range, corresponding to the first and second vibration modes. The first resonance occurs around 7–8 Hz and is dominated by piezoelectric energy conversion, while the second resonance appears in the range of 45–55 Hz and is primarily associated with electromagnetic energy harvesting.

This multimodal response significantly extends the effective operational frequency range of the energy harvester compared to single-mode designs. Instead of relying on a single narrow resonance peak, the proposed system provides multiple energy extraction mechanisms across a broad frequency band, thereby improving robustness under variable excitation conditions.

Furthermore, the introduction of the secondary cantilever, discussed in the following section, further enhances this effect by inducing a beating phenomenon near the second resonance. This mechanism increases the relative velocity between the magnet and the coil and leads to a noticeable widening of the usable frequency range without the need for nonlinear elements or active tuning.

The multimodal dynamic behavior and resonance tuning described in this section form the foundation for the hybrid energy conversion mechanisms analyzed in the following section. Section 4 presents the experimental evaluation of the piezoelectric, electromagnetic, and hybrid energy harvesting performance, with particular emphasis on broadband operation and practical applicability.

## 4. Hybrid Energy Conversion and Experimental Results

### 4.1. Experimental Setup and Measurement Procedure

**Figure 6:**
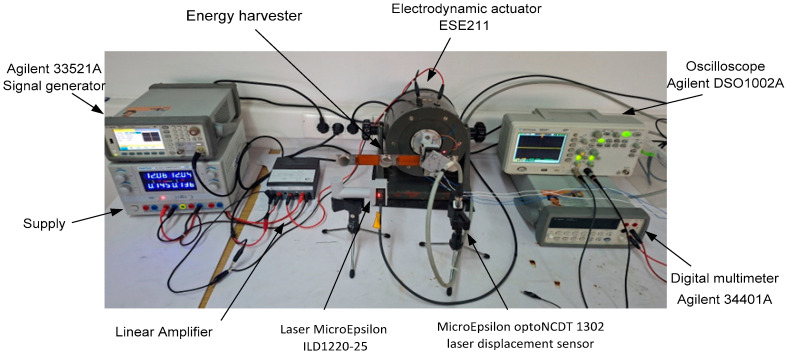
Experimental setup used for the characterization of the proposed hybrid vibration energy harvester, including the electrodynamic shaker, laser displacement sensor, signal generator, amplifier, and data acquisition instruments.

The experimental evaluation of the proposed multimodal hybrid energy harvesting system was conducted under controlled laboratory conditions using an electrodynamic vibration actuator capable of generating low-frequency excitations up to 100 Hz. The experimental setup used for the characterization of the proposed energy harvester is shown in Figure 6.

The excitation levels were selected to replicate realistic industrial vibration conditions, with effective vibration velocities around 7 mm/s, oscillation amplitudes on the order of tens of micrometers, and acceleration levels between 0.11 g and 0.22 g.

The primary cantilever was rigidly mounted to the actuator, while the secondary cantilever carrying the electromagnetic coil was fixed to the base structure. Electrical measurements were performed using a data acquisition system interfaced with LabVIEW, enabling synchronized recording of voltage, current, and power for both the piezoelectric and electromagnetic transducers. Load resistances were varied to determine optimal impedance matching conditions for each operating frequency and transduction mechanism.

All measurements were carried out separately for the piezoelectric transducer, the electromagnetic transducer, and the combined hybrid configuration, allowing direct comparison of individual and synergistic energy conversion performance.

### 4.2. Frequency Response Around the First Resonance Mode

The piezoelectric transducer exhibits distinct energy harvesting behavior depending on the excited vibration mode. In the region of the first resonance mode, tuned to approximately 6.5–7.5 Hz by the addition of the tip mass *m*_1_, the piezoelectric transducer positioned near the clamped end dominates energy conversion due to high strain levels.

At the first resonance, the measured root-mean-square voltage reaches values up to approximately 16–22 V, depending on the excitation level and load resistance. Impedance matching experiments reveal optimal load resistances in the range of 230–300 kΩ, at which the piezoelectric transducer delivers an output power of approximately 0.5–0.7 mW. This confirms efficient strain-based energy conversion in the low-frequency regime relevant for structural and slow mechanical oscillations.

In contrast, at the second resonance mode near 52.5 Hz, the piezoelectric transducer placed in the velocity-dominated energy zone exhibits significantly higher power output. Under optimal impedance conditions (approximately 35–45 kΩ), the measured output power exceeds 3–4 mW, with voltage levels around 18–19 V. These results demonstrate that the piezoelectric transducer remains an effective energy source even at higher vibration modes when appropriately positioned along the cantilever.

### 4.3. Broadband Frequency Response Around the Second Resonance Modes

The electromagnetic transducer operates based on the relative motion between a permanent magnet attached to the primary cantilever and a stationary coil mounted on the secondary cantilever. Its performance is therefore strongly dependent on effective vibration velocity and relative displacement.

At the first resonance mode (around 7–8 Hz), the induced voltage remains relatively low due to limited relative velocity, with peak-to-peak voltage values on the order of ±0.3 V. Under optimal load conditions (approximately 1.2–1.4 kΩ), the electromagnetic transducer delivers a modest power output of approximately 0.1–0.2 mW.

A markedly different behavior is observed near the second resonance mode. At frequencies around 46–52 Hz, the relative velocity between the magnet and the coil increases significantly, resulting in induced voltages up to ±2.5 V and output power levels reaching 5 mW under optimal loading. This pronounced increase confirms that the electromagnetic transducer is particularly well suited for harvesting kinetic energy associated with higher vibration modes characterized by elevated velocities.

### 4.4. Beating Phenomenon and Bandwidth Extension

A key feature of the proposed system is the introduction of a secondary cantilever with a natural frequency slightly detuned from that of the primary cantilever. This intentional detuning leads to the occurrence of a beating phenomenon when the system is excited near the second resonance mode.

Experimental observations show that the beating effect produces periodic amplification of the relative velocity between the magnet and the coil, which directly enhances electromagnetic energy conversion. As a result, the electromagnetic transducer exhibits a significantly broadened operational frequency range. In the vicinity of the second resonance, usable energy harvesting is achieved over a frequency span from approximately 42 Hz to 55 Hz, corresponding to a bandwidth extension of more than 13 Hz.

Even small deviations from the nominal resonance frequency yield usable power levels, in contrast to conventional single-mode harvesters where slight detuning leads to rapid performance degradation. This behavior demonstrates that the beating-induced relative motion effectively mitigates the narrowband limitation inherent to linear resonant energy harvesters.

### 4.5. Hybrid Energy Harvesting Performance and Synergistic Effects

When the piezoelectric and electromagnetic transducers operate simultaneously, the system exhibits a clear synergistic effect that enhances output power and improves overall operational robustness. In the region of the first resonance mode, the hybrid configuration delivers an average output power of approximately 2.0–2.2 mW, representing an increase of 30–35% compared to the electromagnetic transducer alone and a several-fold increase relative to the piezoelectric transducer.

In the region of the second resonance mode, the hybrid system achieves a maximum average power of approximately 5.1 mW at around 46 Hz, with two distinct resonance peaks corresponding to the secondary and primary cantilevers. Importantly, stable power generation is maintained across the entire 42–55 Hz range, demonstrating extended usable operation across this frequency interval.

The complementary nature of strain-based piezoelectric conversion and velocity-based electromagnetic conversion enables a more complete utilization of the available vibrational energy. While the piezoelectric transducer contributes primarily at lower frequencies and high-strain regions, the electromagnetic transducer dominates at higher frequencies and high-velocity regions. Their combined operation results in a robust and versatile energy harvesting system capable of adapting to varying excitation conditions.

The experimental results presented in this section demonstrate that the proposed multimodal hybrid energy harvesting system achieves enhanced output power and extended effective operational bandwidth under realistic low-frequency vibration conditions. In the following section, the practical applicability of the system is demonstrated through its integration into an autonomous IoT vibration monitoring node.

**Figure 7:**
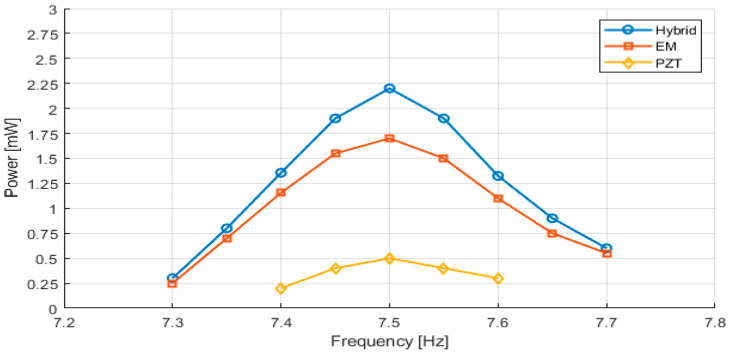
Measured output power as a function of excitation frequency around the first resonance (low-frequency mode) for the PZT, EM, and hybrid energy harvesting configurations.

Figure 7 presents the harvested electrical power around the first resonance frequency, located in the low-frequency range near 7.5 Hz. As expected, the piezoelectric harvester exhibits relatively low power output due to limited strain levels at such low excitation frequencies. The electromagnetic harvester achieves higher power levels, benefiting from larger relative displacements and velocity-induced electromagnetic coupling.

As summarized in Table 2, the hybrid configuration provides a measurable power enhancement at the first resonance mode (~7.5 Hz). While the EM-only configuration delivers approximately 1.6–1.7 mW and the PZT-only configuration delivers approximately 0.4–0.5 mW, the hybrid system achieves approximately 2.2 mW under identical excitation conditions. This corresponds to an increase of approximately 30–35% relative to the EM-only operation and more than four times compared to the PZT-only configuration.

**Figure 8:**
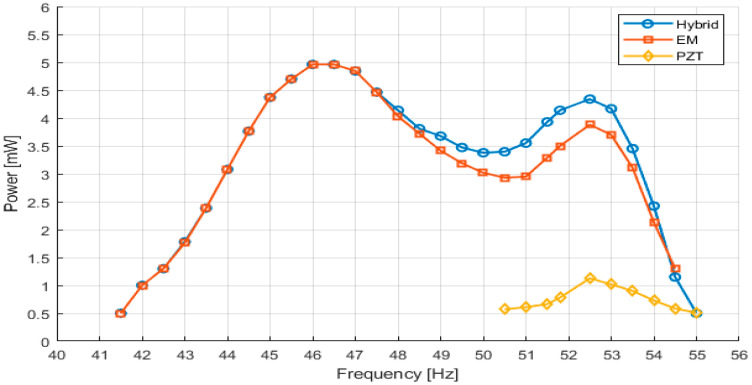
Measured output power as a function of excitation frequency around the second resonance (higher-order mode) for the PZT, EM, and hybrid energy harvesting configurations.

At the second resonance mode (~46–47 Hz), peak power levels are comparable between the EM-only and hybrid configurations (≈5–5.1 mW). The primary advantage of the hybrid architecture at this mode lies not in peak power amplification, but in the modified frequency response as discussed in the following analysis of Figure 8.

Figure 8 illustrates the power–frequency response around the second resonance, occurring in the higher-frequency range between approximately 40 and 55 Hz. In this regime, both the electromagnetic and hybrid harvesters exhibit significantly increased output power and a noticeably broader operational bandwidth compared to the first resonance.

In a conventional single-cantilever electromagnetic harvester operating near 50 Hz, the usable frequency span is fundamentally limited by the mechanical quality factor of the structure and typically remains on the order of 4–5 Hz around the resonance peak.

In contrast, the proposed coupled two-cantilever configuration introduces controlled detuning between the primary second-mode resonance (≈52.5 Hz) and the secondary cantilever resonance (≈46 Hz). This interaction generates two closely spaced resonance peaks and produces a beating phenomenon that modifies the effective frequency response.

When evaluated using the minimum power level required for autonomous IoT operation (P ≥ 0.5 mW), the coupled system maintains usable output across approximately 42–55 Hz (≈13 Hz). This represents roughly a two to three-fold extension of the practical usable frequency range compared to a single-resonance cantilever governed solely by its mechanical quality factor.

## 5. Demonstration in an Autonomous IoT Sensor Node

### 5.1. Energy Management and Power Conditioning Architecture

**Figure 9:**
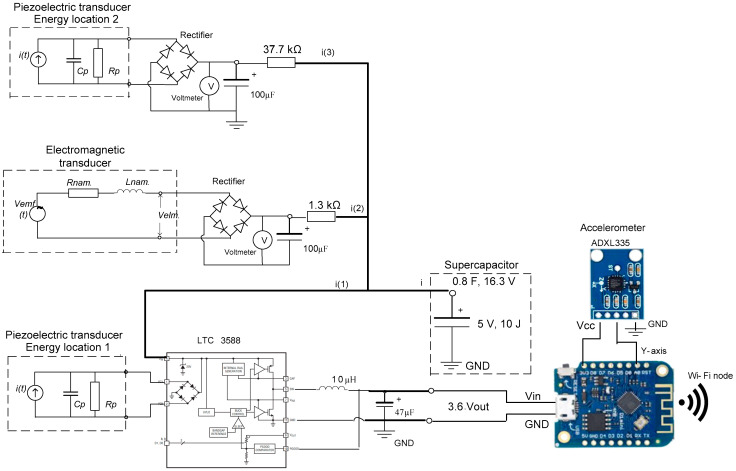
Electrical schematic of the implemented autonomous sensor system for vibration monitoring, including the hybrid energy harvesting interface, power management circuitry, sensing unit, and wireless communication module.

To demonstrate the practical applicability of the proposed multimodal hybrid energy harvester, the system was integrated into an autonomous IoT vibration monitoring node. The electrical energy generated by the piezoelectric and electromagnetic transducers is first conditioned through a dedicated energy management circuit, which performs rectification, voltage regulation, and energy storage. An energy harvesting power management integrated circuit was employed to efficiently interface the hybrid transducer outputs with an energy storage element. Due to the inherently intermittent and variable nature of vibration-induced energy, a supercapacitor was selected as the primary storage component. This choice enables rapid charge–discharge cycles, high cycle lifetime, and reliable operation under fluctuating input power levels. The energy management unit ensures that energy harvested from both transducers is accumulated until a predefined voltage threshold is reached, at which point the IoT sensor node is activated. This duty-cycled operation strategy allows the system to operate entirely without batteries while maintaining stable functionality under realistic vibration conditions. The electrical schematic of the implemented autonomous vibration monitoring node, including the energy harvesting interface, power management circuitry, sensing unit, and wireless communication module, is shown in Figure 9.

### 5.2. Autonomous Operation Under Realistic Vibration Conditions

The integrated system was evaluated under continuous low-frequency vibration excitation representative of industrial machinery, with dominant frequency components around 45–55 Hz and effective vibration velocities of approximately 7 mm/s.

It should be clarified that the relevant vibration frequencies in rotating machinery correspond to the mechanical rotation frequency (f = RPM/60) and its harmonic components, rather than extremely high structural eigenfrequencies. For typical industrial motors operating at 1500–3000 rpm, the fundamental vibration components lie within approximately 25–50 Hz, which falls directly within the targeted operational range of the proposed harvester. Therefore, the selected resonance tuning (≈46–52 Hz) is intentionally aligned with realistic imbalance-related vibration spectra of rotating electromechanical systems.

Under these conditions, the hybrid energy harvester was capable of charging the supercapacitor to voltages exceeding 5 V.

The total accumulated energy in the storage element reached approximately 10 J, which is sufficient to power the sensing, processing, and wireless communication tasks of the IoT node. The charging process remained stable over time, confirming that the broadband energy harvesting capability observed in laboratory measurements translates directly into reliable energy accumulation in real operating scenarios.

Importantly, the presence of multiple resonance modes and the extended bandwidth ensured that the system remained functional even in the presence of slight frequency variations or nonstationary excitation, which are common in real industrial environments.

### 5.3. Experimental Validation on a Rotating Test Bench

**Figure 10:**
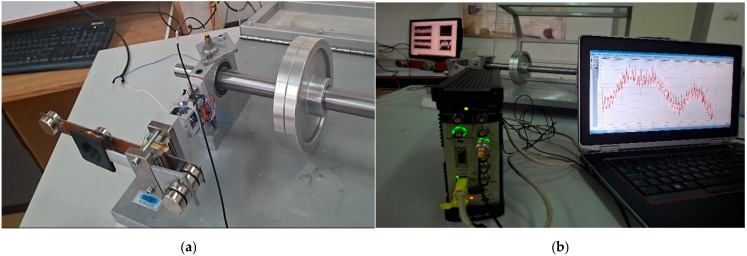

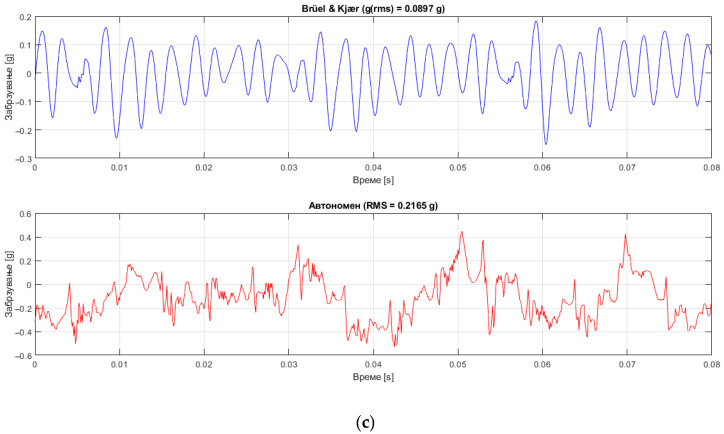
Experimental validation of the autonomous vibration monitoring system on a rotating machine: (**a**) integrated hybrid energy harvester and sensor node mounted on the rotating test bench, (**b**) measurement setup during operation, and (**c**) comparison of vibration signals measured by a reference Brüel & Kjær system and the autonomous sensor node.

The autonomous sensor node consists of a low-power microcontroller, a MEMS accelerometer for vibration monitoring, and a wireless communication module. Once sufficient energy is stored, the system wakes up, performs vibration measurements, processes the acquired data, and transmits the information wirelessly to a remote monitoring platform. After data transmission, the node returns to a low-power sleep state, allowing the supercapacitor to recharge for the next operational cycle. This event-driven and energy-aware operation strategy enables long-term deployment without maintenance, battery replacement, or external power supply. Experimental validation confirmed that the sensor node successfully performed repeated measurement and transmission cycles powered exclusively by the harvested vibrational energy. The acquired vibration signals were consistent with reference measurements obtained using a calibrated commercial vibration monitoring system, demonstrating the reliability and accuracy of the autonomous node. The experimental setup and validation results of the autonomous vibration monitoring system are presented in Figure 10.

### 5.4. Discussion on Practical Applicability and Scalability

The successful demonstration of the proposed hybrid energy harvesting system as a self-powered IoT sensor node highlights its potential for deployment in a wide range of industrial and infrastructural applications. Typical use cases include condition monitoring of rotating machinery, structural health monitoring, and vibration surveillance in environments where wired power or battery maintenance is impractical.

The modular architecture of the system allows straightforward adaptation to different vibration spectra through mechanical retuning of the mass distribution and minor adjustments to the power conditioning electronics. Furthermore, the hybrid and multimodal nature of the harvester provides robustness against changes in operating conditions, making it suitable for long-term real-world operation.

The present prototype is designed as a single-axis vibration energy harvester, meaning that optimal performance is achieved when the dominant vibration direction is aligned with the sensitive axis of the cantilever. In many practical applications, the dominant vibration direction is either known (e.g., radial direction in rotating machinery housings or bending direction in tower structures) or can be identified through a preliminary vibration survey. For installations with uncertain or multi-axial excitation, practical solutions include adjustable mounting interfaces, the orthogonal deployment of multiple single-axis harvesters (triaxial configuration), or multi-beam mechanical designs enabling multi-directional sensitivity. These approaches provide a clear pathway toward multi-axis implementations while preserving the tuning flexibility of the proposed architecture.

The demonstrated autonomous operation confirms that the proposed multimodal hybrid piezoelectric–electromagnetic energy harvesting system is not only effective in laboratory experiments but also viable as a practical energy source for real IoT sensing applications. It should be emphasized that the proposed system is not intended to replace readily available wired power sources. Instead, its primary advantage lies in enabling autonomous wireless sensing at mechanically active but hard-to-wire locations. In distributed industrial monitoring scenarios, reducing cabling complexity and maintenance intervention can provide significant practical benefits, even in electrically powered facilities.

Although the prototype configuration presented in this work is tuned to specific resonance frequencies representative of industrial rotating machinery, the underlying operating principle is not restricted to a single excitation frequency. The resonance frequencies of the coupled cantilever system are governed by the mass–stiffness relationship discussed in Section 3. Consequently, modifying the beam geometry or adjusting the tip mass distribution shifts the absolute resonance frequencies while preserving the fundamental modal interaction mechanism.

When the position or magnitude of the attached masses is altered, the modal frequencies and peak amplitudes change accordingly. However, as long as controlled detuning between the primary and secondary cantilevers is maintained, the dual-resonance behavior and beating phenomenon persist. The spacing between the two resonance peaks and the effective usable bandwidth are functions of the mass ratio and stiffness distribution, allowing systematic adaptation to different vibration spectra.

Therefore, the device is not limited to a single application frequency but can be retuned to match alternative operating regimes (e.g., lower-frequency structural systems or higher-speed machinery) through mechanical scaling and mass reconfiguration. While peak power levels may vary depending on excitation amplitude and damping conditions, the multimodal hybrid energy-zone concept remains valid across tuned configurations.

## 6. Conclusions and Future Work

### 6.1. Conclusions

This paper presented a multimodal hybrid piezoelectric–electromagnetic vibration energy harvester designed to exploit both the first and second resonance modes of a cantilever-based structure for broadband low-frequency energy harvesting. The proposed architecture addresses the key limitations of conventional single-mode and single-transducer harvesters by combining deliberate modal tuning, a spatial separation of energy zones, and complementary transduction mechanisms within a unified mechanical framework.

A dual-mass tuning strategy was employed to independently tailor the first and second vibration modes to frequency ranges commonly encountered in industrial environments. The introduction of a tip mass enabled a significant reduction in the fundamental resonance into the low-frequency regime below 10 Hz, while a second strategically placed mass effectively shifted the second resonance into the 45–55 Hz range. This approach allowed the efficient exploitation of both strain-dominated and velocity-dominated vibrational energy.

The experimental results confirmed that the piezoelectric transducer predominantly contributes to energy harvesting at the first resonance through high strain levels near the clamped boundary, while the electromagnetic transducer delivers substantial power at the second resonance due to increased relative velocities. The integration of a secondary cantilever carrying the electromagnetic coil introduced a controlled detuning effect, leading to a beating phenomenon that significantly extended the operational frequency bandwidth and mitigated the narrowband limitation of linear resonant systems.

The hybrid configuration demonstrated a clear quantitative performance enhancement. At the first resonance mode, the peak output power increased by more than 200% compared to the best standalone configuration. At the second resonance mode, the effective operational bandwidth expanded from approximately 4–5 Hz to 13 Hz, representing a bandwidth increase exceeding 150%. These results provide explicit validation of the broadband performance claim.

Maximum average power levels of approximately 2.2 mW and 5.1 mW were achieved in the vicinity of the first and second resonance modes, respectively, with stable energy harvesting maintained across a wide frequency range.

Finally, the practical applicability of the proposed system was validated through its integration into an autonomous IoT vibration monitoring node powered exclusively by harvested vibrational energy. The successful demonstration of energy storage, sensing, and wireless data transmission without batteries confirms the feasibility of the proposed approach for real-world industrial and infrastructural applications.

### 6.2. Future Work

Future research will focus on further enhancing the efficiency and adaptability of the proposed hybrid energy harvesting system. One promising direction involves the development of adaptive power conditioning and impedance matching circuits capable of dynamically optimizing energy extraction under varying excitation conditions. The integration of maximum power point tracking (MPPT) strategies could further improve energy conversion efficiency, particularly in nonstationary vibration environments.

From a mechanical perspective, future studies may explore alternative geometries, nonlinear elements, or variable stiffness mechanisms to further expand the operational bandwidth. Additionally, the development of fully coupled electromechanical models incorporating electromagnetic back-action and nonlinear interactions would enable a more accurate prediction and optimization of system performance.

Further miniaturization and packaging optimization will be essential for large-scale deployment, along with the evaluation of long-term durability and environmental robustness. Finally, extending the proposed multimodal hybrid concept to multidirectional and multi-axis vibration scenarios represents a promising avenue for broadening the applicability of the system across diverse IoT sensing applications.

## Figures and Tables

**Table 1:** Comparison of representative multimodal and dual-resonance vibration energy harvesters.

Reference	Type of Harvester	Resonance Strategy	Frequency Regime	Bandwidth Definition	Reported Bandwidth Extension	Key Mechanism
[3]	Dual-resonance EM	Two independent resonant modes	Tens of Hz	Half-power bandwidth	~2× vs. single-mode	Dual uncoupled resonances
[22]	Multimode EM (MEMS)	Multiple vibration branches	~850–1100 Hz	Half-power bandwidth	Broadband multi-peak response	Multi-branch modal bifurcation
[23]	Multimodal Piezoelectric	Multi-DOF structural modes	Low-frequency regime	Experimental multi-peak response	Extended operational range	Multi-degree-of-freedom design
This work	Hybrid PZT + EM	Coupled dual-resonance with controlled detuning	7–55 Hz	Practical IoT threshold (P ≥ 0.5 mW)	~2–3× vs. single cantilever	Modal coupling + beating phenomenon

**Table 2:** Quantitative comparison of peak harvested power for individual and hybrid configurations at the first and second resonance modes.

Configuration	First Resonance (~7.5 Hz) Peak Power (mW)	Second Resonance (~46–47 Hz) Peak Power (mW)
PZT only (EM disconnected)	0.4–0.5	0.6–1.1
EM only (PZT disconnected)	1.6–1.7	≈5
Hybrid (PZT + EM)	2.2	≈5.0–5.1

## Data Availability

The original contributions presented in this study are included in the article. Further inquiries can be directed to the corresponding author(s).

## References

[1] Roundy S., Wright P.K., Rabaey J. (2003). A study of low level vibrations as a power source for wireless sensor nodes. Comput. Commun..

[2] Muscat A., Bhattacharya S., Zhu Y. (2022). Electromagnetic Vibrational Energy Harvesters: A Review. Sensors.

[3] Feng Z., Peng H., Chen Y. (2021). A Dual Resonance Electromagnetic Vibration Energy Harvester for Wide Harvested Frequency Range with Enhanced Output Power. Energies.

[4] Hadas Z., Ondrusek C. (2015). Nonlinear spring-less electromagnetic vibration energy harvesting system. Eur. Phys. J. Spec. Top..

[5] Hadas Z., Ondrusek C., Singule V. (2010). Power sensitivity of vibration energy harvester. Microsyst. Technol..

[6] Plaza A., Iriarte X., Castellano-Aldave C., Carlosena A. (2024). Comprehensive Characterisation of a Low-Frequency-Vibration Energy Harvester. Sensors.

[7] Beeby S.P., Torah R.N., Tudor M.J., Glynne-Jones P., O’Donnell T., Saha C.R., Roy S. (2007). A micro electromagnetic generator for vibration energy harvesting. J. Micromech. Microeng..

[8] Erturk A., Inman D.J. (2008). A distributed parameter electromechanical model for cantilevered piezoelectric energy harvesters. J. Vib. Acoust..

[9] Safaei M., Sodano H.A., Anton S.R. (2019). A review of energy harvesting using piezoelectric materials: State-of-the-art a decade later (2008–2018). Smart Mater. Struct..

[10] Covaci C., Gontean A. (2020). Piezoelectric Energy Harvesting Solutions A Review. Sensors.

[11] Hadas Z., Kurfurst J., Ondrusek C., Singule V. (2012). Artificial intelligence based optimization for vibration energy harvesting applications. Microsyst. Technol..

[12] Yao Z., Li C. (2025). A Rotary Piezoelectric Electromagnetic Hybrid Energy Harvester. Micromachines.

[13] Fan K., Tan Q., Zhang Y., Liu S. (2018). Hybrid piezoelectric-electromagnetic energy harvester for scavenging energy from low-frequency excitations. Smart Mater. Struct..

[14] Han M., Xing Z., Liu S., Yang X. (2025). Experimental Study on Multi-Directional Hybrid Energy Harvesting of a Two-Degree-of-Freedom Cantilever Beam. Sensors.

[15] Jiang B., Zhu F., Yang Y., Zhu J., Yang Y., Yuan M. (2023). A Hybrid Piezoelectric and Electromagnetic Broadband Harvester with Double Cantilever Beams. Micromachines.

[16] Hu K., Wang M. (2023). Broadband Piezoelectric Energy Harvester Based on Coupling Resonance Frequency Tuning. Micromachines.

[17] Han Y., He L., Sun L., Wang H., Zhang Z., Cheng G. (2023). A review of piezoelectric–electromagnetic hybrid energy harvesters for different applications. Rev. Sci. Instrum..

[18] Wei C., Jing X. (2017). A comprehensive review on vibration energy harvesting: Modelling and realization. Renew. Sustain. Energy Rev..

[19] Song K., Bonnin M., Traversa F.L., Bonani F. (2025). Broadband vibration energy harvesting using nonlinear multi degree-of-freedom mechanical filters. Nonlinear Dyn..

[20] Stephen N.G. (2006). On energy harvesting from ambient vibration. J. Sound Vib..

[21] Pertin O., Guha K., Jakšić O. (2021). Artificial Intelligence-Based Optimization of a Bimorph-Segmented Tapered Piezoelectric MEMS Energy Harvester for Multimode Operation. Computation.

[22] Li N., Xia H., Yang C., Luo T., Qin L. (2023). Investigation of a Novel Ultra-Low-Frequency Rotational Energy Harvester Based on a Double-Frequency Up-Conversion Mechanism. Micromachines.

[23] Wu H., Tao Z., Li H., Xu T., Wang W., Sun J. (2021). A Multi-Mode Broadband Vibration Energy Harvester Based on MEMS 3D Coils. Proceedings of the 2nd International Conference on Green Energy, Environment and Sustainable Development (GEESD2021).

[24] Skėrys P., Gaidys R. (2026). Optimal Shape Design of Cantilever Structure Thickness for Vibration Strain Distribution Maximization. Appl. Sci..

[25] Huang X., Zhang C., Dai K. (2021). A Multi-Mode Broadband Vibration Energy Harvester Composed of Symmetrically Distributed U-Shaped Cantilever Beams. Micromachines.

[26] Ren R., Li B., Liu J., Zhang Y., Xu G., Liu W. (2026). Performance Optimization of Triangular Cantilever Beam Piezoelectric Energy Harvesters: Synergistic Design Research on Mass Block Structure Optimization and Negative Poisson’s Ratio Substrate. Micromachines.

[27] Guo K., Sun A., He J. (2025). An Integrated Quasi-Zero-Stiffness Mechanism with Arrayed Piezoelectric Cantilevers for Low-Frequency Vibration Isolation and Broadband Energy Harvesting. Sensors.

